# Impact of music learning on students’ psychological development with mediating role of self-efficacy and self-esteem

**DOI:** 10.1371/journal.pone.0309601

**Published:** 2024-09-03

**Authors:** Jing Jiang

**Affiliations:** School of Music & Dance, Zhengzhou Normal University, Zhengzhou, Henan, China; The Open University of Israel, ISRAEL

## Abstract

Professionals and academics have recently placed a greater emphasis on comprehending the elements that go into improving students’ psychological wellbeing. Students frequently face major obstacles as a result of the rigorous nature of academic life, which can result in problems like tension, depression and other psychological health concerns. These complications have a long-lasting influence on their future aspirations in addition to affecting their academic achievement. This study determined the effects of music learning on students’ academic performance and psychological well-being. The mediating role of self-efficacy and self-esteem are also examined in this study. The data is collected from 326 students in Chinese universities and applied structural equation modeling for empirical analysis. The findings show that music education improves the students’ psychological well-being, which in turn improves their academic performance. Additionally, psychological health is a major factor in improving the academic performance. There is significant mediating impact of self-efficacy and self-esteem in relationship between mental well-being and music education. To improve students’ psychological health, it is suggested that policy makers should consider the integration of music education into academic settings.

## 1. Introduction

The academic research demonstrates the significant impact of the music education on people’s mental and behavioral health [1, 2], while some studies [3] make different claims. The uncertainty around the social lives, careers, and academic achievement, university students are among those, most negatively affected by these circumstances [4]. The anxiety, depressive attitudes, stumpy self-esteem, psychiatric disorders, substance infatuation, and hopeless thoughts and actions are becoming more common among students globally [5–7]. As a result, students nowadays have need of more support for the health conditions. To facilitate the students successfully, university professionals must implement predetermined steps to mitigate the detrimental effects on psychological well-being. These outcomes highlight the need of establishing suitable approaches to address the health problems of students [8]. Historically, university students have underutilized mental and counselling facilities. Identifying the populations impacted by psychological impacts can lead to more targeted therapies, successful treatment, and coping methods for the most vulnerable. The government applied a variety of steps to avoid the spread of health issues in students [9–14].

Mental health of students is a complex and multifaceted issue, influenced by various factors including academic pressure, societal expectations, economic stress, and cultural norms. The competitive nature of education system places significant pressure on students to excel academically. “This pressure can lead to stress, anxiety, and depression among students striving to meet high expectations” [15]. Many students face challenges adjusting to university life, making new friends, and managing increased independence. Despite growing awareness, mental health issues are still stigmatized in Chinese society. This can prevent students from seeking help due to fear of being judged or discriminated against [16, 17]. Cultural expectations, particularly regarding filial piety and academic achievement, can create additional stress for students who feel pressure to meet these expectations [18]. Some students face financial difficulties, either in supporting themselves through university or in meeting family expectations for financial contributions [19]. Efforts to address mental health among university students include increasing mental health awareness, providing more accessible and affordable mental health services [20, 21].

The purpose of this study is to analyze a relation among music learning, academic performance and well-being of university students. Developing socially competent, creatively thinking individuals, are a core goal of high-quality education. Music education has the power to ignite students’ desires, emotions, and other qualities related to stimulate the imagination and vision, which can help students to develop their creative identities. Therefore, music education programs at universities are a useful tool for helping students to overcome "poorly functioning" personalities [22]. By teaching music, educators may help the students to develop their creative identities and boost their self-efficacy, which can help them overcome personality flaws that have been made worse by a number of unfavorable characteristics of the current cultural contexts [23]. Academic curriculum that incorporate music education are thought to encourage students’ creative thinking. This pedagogical strategy might begin with theoretical discussions on the effect of music on psychological processes for benefit of students. Moreover, a blended learning strategy might improve students’ mental health by fusing this theoretical framework with effective teaching techniques. Promoting active participation in music education among students may help them to improve their mental well-being and academic performance. Students’ interpersonal communication skills would improve as a result of this engagement, which would also help to develop their open-mindedness. This would promote a culture of social understanding and strengthen the students’ feelings of self-control. Additionally, taking part in music education has been linked to improved psychological wellness and decreased anxiety, both of which support general sound health [24].

Mostly people agree that learning music is not just a way to acquire a skill; it also plays a significant part in treating and regulating psychological issues [25]. Therefore, since developing students’ personalities is the main objective, adding music education into academic institutions may result in a special role being played in resolving the psychological disorders. On the other hand, some music teachers could occasionally use intimidating strategies and overburden the students with knowledge [26]. It might be challenging for students to keep up the pace of studying this area, even once external factors are taken out of the picture. As a result, institutions have to give a lot of attention for students’ mental health and usage of music education techniques. These steps are important for psychological control and are frequently used in practice of providing mental health care [27]. Providing music education rooms that are furnished with instructional resources can be especially helpful in improving the psychological fitness of the learners. This technique might support the students in recognizing the value of cognitive skills in supporting learning by offering pertinent elective courses in music psychology that align with their educational goals and personal experiences. It may help the students to create supportive social networks and improve peer relationships [28]. In addition, using the internet to provide students with digital psychological counseling activities, suggesting interesting and inspiring music compositions, and sharing basic music education information may all help the students to find psychological satisfaction in music. In addition, the universities can treat psychological issues by using music as a tool to improve student-to-student contact through concerts, games, and other events. By gently monitoring students’ self-awareness about study stress, helping one another to lessen despair, and fostering an environment of support, collaboration, and affection, this strategy seeks to promote overall psychological well-being [29, 30]. Well-established ideas like self-efficacy and self-esteem may have an impact on how music education affects students’ academic performance, psychological health, and overall well-being. Self-efficacy is the trust that one can control one’s surroundings and so influence one’s behavior, viewpoints, and expectations for the future. An individual’s total assessment of their own value, whether good or negative, is referred to as their self-esteem. It has been interconnected to happiness, contentment, and efficient stress management [31, 32]. Within the framework of this research, it is anticipated that both self-efficacy and self-esteem would provide noteworthy consequences, acting as intermediaries in the connection between music instruction and students’ educational performance and their mental well-being. The study has the following objectives to better understand the connections between students’ wellbeing and performance: (1) To examine the relation among students’ psychological wellbeing and academic performance; (2) To determine the mediating role of self-esteem in the relationship among well-being and academic performance; and (3) to find the role of self-efficacy as a mediator in the relationship among psychological wellbeing and music education and academic performance.

There are four primary components of the research. An introduction and a review of pertinent literature are included in the first part. The formation of hypotheses and the creation of the research model are the main topics of the second portion. The third section goes into detail about data analysis and research technique. Lastly, the discussion of the results and closing thoughts are included in the fourth part.

## 2. Literature review and hypothesis development

The objective of this research is to investigate how music education affects students’ academic achievement and psychological health. It also examines the ways in which self-efficacy and self-esteem act as a mediating variable in this relationship. The theories listed below provide credence to these investigations.

### 2.1. Theory of social cognitive

This theory clarifies that how humans work by emphasizing on interaction processes. A substantial importance is given to cognitive processes, which enable people to learn from their surroundings. People regulate their self-efficacy processes by considering and integrating their own thoughts and practices. This integration of this theory with music training/learning aims to consider the importance of enlargements in creating efficient teaching and learning strategies for students. When creating curriculum and instructional services for individuals, it is important to have a strong theoretical basis in order to comprehend how learning happens. In order to describe how humans work, social cognitive theory places an emphasis on a dynamic, interacting process incorporating behavioral, personal, and environmental elements. A system of triadic reciprocal causations characterizes this perspective of human interactions. The theory emphasizes the significance of cognitive processes, in which people watch other people, consider these observations in concurrence with particular thoughts [33]. It makes sense to consider learning therapies for this population when applying a learning model that emphasizes the significance of cognition. According to the social cognitive paradigm, perceived self-efficacy and human agency are two key components that influence cognitive development and performance. Thus, a link may be found between this theory and how music education helps students develop certain cognitive skills for psychological well-being.

### 2.2. Self-esteem theory

“Self-esteem remains one of the most researched issues in social psychology. Even though it is frequently seen as a part of one’s self-concept, a lot of people give it a lot of weight” [34]. In the past, self-esteem and self-concept have occasionally been used interchangeably in literature. Self-esteem has received a lot of attention because of the strong link with a number of outcomes for both individuals and societies. Furthermore, it’s well accepted that raising one’s self-esteem is good for society and the person, especially for kids and teenagers [35]. A person’s general sense of self or certain components of it, such as their opinions about their social standing, race or ethnicity, physical characteristics, athletic prowess, and academic or professional accomplishments, can all be included in their self-esteem. In fact, many forms of self-esteem have been classified by theorists into categories like contingent versus non-contingent, visible versus tacit, real versus false, stable versus volatile, and global versus domain-specific. Many academics perceive the complexity of self-esteem as a single, all-encompassing quality, while others regard it as a heterogeneous quality made up of several elements including social, cognitive, and factual self. A distinction has been made amid an inauthentic self-perception and an authentic self-perception. “Contingent self-esteem” is defined as self-esteem that depends on relationship or psychological values, or on achieving certain success standards [36]. This kind of self-worth is associated to being ego-involved in goals and working hard to accomplish them. A typical correlation between contingent self-esteem and narcissism is social comparison. The study evaluated the learning satisfaction of Brazilian students and found that “the students of private schools and earlier academic years were the ones who obtained the most satisfaction with the study techniques and with the infrastructure. These results provide greater knowledge about the processes of self-regulation and external regulation of university learning and of their satisfaction with them, which can contribute to improving educational policies in Brazil” [34]. As essential components of their self-concept, action, proactivity, and vitality are how people show their worth. Many academics make a distinction between implicit and explicit self-esteem when evaluating self-esteem; the latter refers to the introspective but unacknowledged impact of one’s own attitude on assessments of oneself in comparison to detached things. Within the framework of this hypothesis, the association between academic outcomes and music education was found to be mediated by self-esteem.

### 2.3. Self-determination theory

“The ability of a person to make choices and run their own life is known as self-determination, and it is a basic psychological notion” [19]. This skill is necessary for mental health and well-being in general. People with self-determination mindsets feel in control of their life, which motivates them to act when they think their actions will have an effect on the solution. The notion of self-determination is given to academicians Deci and Ryan as pioneers [37]. The book "Self-Determination and Intrinsic Motivation in Human Behavior" has a full account of their research. According to a motivational hypothesis put out by Ryan and Deci [38], people are largely motivated by a desire to better themselves. This theory, which emphasizes psychological well-being, is called self-determination theory. It covers motivation and personal growth. This concept holds that people are naturally curious and have a voracious appetite for information, and that one’s sense of well-being, capacity for self-control, and innate drive to learn may all be positively or negatively influenced by their surroundings. According to the idea, three essential psychological demands are competence, autonomy, and belonging. The study [38] on music involvement built upon this notion. Theoretical foundations gave the mediators pertaining to students’ psychological well-being and educational attainment in this study a strong basis.

### 2.4. Relationship among psychological health, self-esteem, self-efficacy and music education

Many researchers [38–41] have examined the impact of music learning on psychological wellbeing of students from a variety of perspectives. Music education may foster students’ creative personalities by igniting their imaginations and associations, which can further boost their benefits, and non-intellectual features. As such, inclusion of music education in university curriculum might help individuals overcome whatever "dysfunctional" parts of themselves. With the help of music education, students may control who they are and develop a feeling of self-efficacy that helps them overcome personality defects brought on by the constraints of modern culture [22]. By seeing music education as a type of creative education with implications for psychological control and therapy, educational institutions may successfully utilize the therapeutic potential of music education in treating mental health challenges. The advantages of music education for personal development are still substantial even if they are not given as much attention. Research on the relationship between learning music and developing psychological benefits, such as self-efficacy, is still ongoing. A study examined the middle and high school children who participate in band, chorus, and orchestra programs in terms of their self-efficacy. The outcomes showed a little optimistic relationship between self-efficacy and melodious ability [42, 43]. Students who were more proficient musicians also showed sophisticated levels of self-efficacy. But present research work study looks at the association among self-efficacy, self-esteem, and music education. Although students’ skills, musical aptitude, and cognitive abilities may be similar, empirical data indicates that children who receive paid piano lessons have notable advantages in the enlargement of their self-esteem when compared to children who do not have access to music education tutoring [44, 45].

The study [46] focused on the music curriculum that elementary school students participated during the school days. Self-esteem declined in the non-musically control group but did not diminish in the musical learning group. Children who participated in a hand beating music platform in Australia showed a considerable improvement in self-esteem after completing the program [47]. Investigators that looked at high school students’ motivations for taking part in extracurricular music activities discovered that many of them felt more confident and self-sufficient. Similar to this, African drumming enthusiasts felt that they were furthering the cause of music, which increased their feeling of self-efficacy and contentment with their scholastic endeavors [48]. All of these research shows the relation among mental health, self-efficacy, and self-esteem among students who get music education. Consequently, we suggest the following:

***H1*:** There is a positive relationship between psychological well-being and music education in university students.
***H2*:** Self-esteem and music learning have a positive relationship in university students.
***H3*:** Self-efficacy and music learning have a positive relationship in university students.

### 2.5. Self-esteem and psychological well-being

Studies on the topic of self-esteem reveal a noteworthy correlation among psychosomatic well-being and self-esteem. Nevertheless, the nature of the association changes according to the kind of self-esteem being considered. Research has consistently shown that improving psychological well-being is positively impacted by self-esteem [49]. Furthermore, it has been determined that culture has an impact on the causal link between happiness and self-esteem. It has been discovered that, in contrast to collectivist civilizations, self-esteem is more closely linked to life satisfaction in individualistic cultures. Each cultural setting has its own set of values and priorities, which give birth to this variance. Individuals in idiosyncratic the social order are more probable to value their individuality and distinctive characteristics, which increases the importance of self-esteem. Conversely, though, relational and community qualities of the self are valued more than individual characteristics in collectivist civilizations. Understanding the kinds of self-esteem that promote psychological well-being is especially important in collectivist societies [49, 50]. By shifting their attention from individual self-esteem to the social level, numeral research studies have inspected the connection among psychological well-being and self-esteem [23, 36]. The influence of collective self-esteem on individuals’ well-being was examined in these research, and found that the collective self-esteem is essential in forming self-perception. Remarkably, research showed that among American kids that identify as White, Black, or Asian, there is a strong relation between well-being and self-esteem. The connotation amongst collective self-esteem and well-being differed throughout ethnic groups, though, when a person self-esteem was reserved into account. This linkage remained tiny for Black students, became insignificant for White learners, and grew moderate to substantial for Asian students. This demonstrates the profound impact of culture on the significance of different types of self-esteem [51]. There is a robust association among students’ self-esteem and well-being, as shown by a number of research [52]. Furthermore, a number of research investigations have recognized the facilitating function of self-esteem from diverse angles and have demonstrated its noteworthy involvement as arbitrator [53, 54]. In light of our study’s particular setting, these results imply that self-esteem plays an intermediating role in association among psychological health and music education, which prompts us to formulate the following theories:

***H4*:** There is a positive relationship between psychological health and self-esteem.
***H5*:** Self-esteem acts as a mediating factor in the link between psychological well-being and music education.

### 2.6. Self-efficacy and psychological well-being

Individuals who possess higher self-efficacy demonstrate a proactive mindset, viewing problems as opportunities to achieve instead of things to avoid. They establish high standards for themselves and show tenacity in reaching them. Those who possess high self-efficacy are actively involved in life and, in the face of difficulty, remain optimistic about their capacity to handle the circumstance. In another hand, lower psychological well-being and enlarged indications of unease and downheartedness have been linked to low self-efficacy [55]. Earlier research work [56] has established a robust optimistic association between psychological well-being and self-efficacy, which has led to more research on this relationship in the perspective of present study. Furthermore, a number of investigators have discovered circumstances in which self-efficacy acts as a moderator, exerting a significant influence amongst altered factors and situations [57–59]. The following hypotheses for this study have been developed in light of these findings, which demonstrate the significant positive mediation of self-efficacy:

***H6*:** Psychological well-being improves the self-efficacy of students.
***H7*:** Self-efficacy mediates the relation among musical education and psychological well-being.

### 2.7. Relationship between psychological wellbeing and academic performance

Earlier studies have explored the link among students’ psychological health and their academic performance, yielding significant findings [56, 60–64]. Among university students globally, psychological suffering has become a major and pressing problem. Five of the upper six health relatable worries of students were found to be psychological in nature, according to a US survey. Students with high psychological distress and poor psychological well-being are the two groups into which students with severe mental illness can be divided. In a study, Australian university students discovered that while low psychological well-being was linked to more despair, good psychological well-being was tied with lower levels of sadness. The research employed a scale to appraise students’ mental well-being and distress, clarifying the connection between the two variables [65–67]. We have put forth the following hypothesis in light of the supporting research, which indicates a relationship between students’ performance and well-being in the classroom:

***H8*:** Better psychological health of students improve their academic performance.

## 3. Method

### 3.1. Participants

The human research ethics committee of Zhengzhou Normal University approved the study with reference number ZNU/HEC/2023/786 on November 20, 2023. A written consent from the participants is gained prior to collect the data. The data was collected from November 27, 2023 to February 29, 2024. Students enrolled in Chinese universities are the main focus of this research study. A survey questionnaire was created and distributed to university students to collect the data. We received the 348 of the 400 surveys that were sent, yielding an 87% response rate. Moreover, 326 survey were selected for final empirical analysis as those questionnaires were dropped, having some ambiguities or incomplete. The survey was sent via online resources, like emails, social media etc. A convenience sample strategy was used in this investigation because convenience sampling is so simple to use and earlier literature also supports this method of sampling [68, 69]. Table 1 displays the general characteristics of the respondents of the survey.

**Table 1:** Characteristics (N = 326).

	Frequency	Percentage
Gender		
Male	171	52.45
Female	155	47.55
Age		
18–20	82	25.15
21–23	196	60.12
24 and Above	48	14.72
Education		
Undergraduate	176	53.99
Graduate	150	46.01
Field of Study		
Social Sciences	102	31.29
Natural Sciences	56	17.18
Engineering	49	15.03
Art and Music	119	36.50
Residence Area		
Rural	144	44.17
Urban	182	55.83

### 3.2. Instruments

Twenty-five items were selected from the literature to be included in the questionnaire for this study. Psychological well-being is measured with a 5-item scale adapted from the study [51]. The authors of the study proposed a multi-dimensional model of well-being, encompassing the autonomy- feeling independent and self-directed; environmental mastery- ability to manage and shape one’s environment; personal growth- continuously developing and improving; positive relations- having warm and supportive relationships; purpose in life—having a sense of direction and meaning; and self-acceptance. By using these measures, the study assessed the psychological well-being as a comprehensive and multi-faceted construct. Music education was assessed using a five-item used by the study [70] which referred to the cognitive, emotional, and behavioral dispositions that enable students to engage in intelligent and creative thinking. The researchers developed a scale to measure the music education, consisting of analyzing and evaluating musical information, taking responsibility for one’s own music learning, working effectively with others in music-making, recognizing and managing emotions in music experiences, overcoming obstacles and challenges in music learning, and embracing diverse musical perspectives and styles. A comprehensive scale is created to assess music education, comprising five key dimensions.

1. Analyzing and Evaluating Musical Information
  This dimension measured the students’ ability to identify and explain musical elements (e.g., melody, harmony, rhythm), analyze the musical structures and forms, evaluate musical performances or compositions, and demonstrate critical thinking skills in music.
2. Taking Responsibility for One’s Own Music Learning:
  This dimension assessed the students’ ability to set goals and prioritize tasks for music learning, practice effectively and manage time, seek feedback and reflect on their own learning, demonstrate self-motivation and discipline in music learning.
3. Working Effectively with Others in Music-Making
  This dimension evaluates students’ ability to collaborate with peers in music ensembles or groups, communicate effectively with others about music, support and respect fellow musicians, demonstrate teamwork and leadership skills in music-making.
4. Recognizing and Managing Emotions in Music Experiences
  This dimension measures students’ ability to identify and express emotions through music, recognize emotional responses to music, manage performance anxiety or stress, demonstrate empathy and understanding of others’ emotional responses to music.
5. Overcoming Obstacles and Challenges in Music Learning
  This dimension assessed students’ ability to persist through difficulties or frustrations in music learning, seek help and resources when needed, adapt to new or challenging musical situations, demonstrate resilience and grit in music learning.

By assessing these dimensions, the scale provided a comprehensive picture of students’ music education, encompassing cognitive, affective, and behavioral aspects of music learning. Furthermore, a five-item self-efficacy measure adapted from the study [71] with following constructs. “I can always manage to solve difficult problems; If someone opposes me, I can find the means to get what I want, It is easy for me to stick to my aims and accomplish my goals, I am confident that I can handle unforeseen situations, I can remain calm when facing difficulties because I can rely on my coping abilities.” A four-element self-esteem scale taken from Rosenberg [72] were used for the assessments. Participants rated their agreement with statements such as "I feel that I’m a person of worth, at least on an equal plane with others, I feel I do not have much to be proud of, I have a positive attitude toward myself, I am able to do things as well as most other people." A six-element scale that was modified from the study [73] was employed to measure the performance of students using the following indicators; Grade Point Average (GPA), academic achievement scores in specific subjects, students’ performance on standardized tests, like quizzes and exams, students’ ranking in their class based on their overall performance, teachers’ evaluations of students’ performance. These indicators provide a comprehensive picture of students’ academic performance. This research uses a cross-sectional, quantitative methodology. Smart-PLS was utilized to employ the partial least squares (PLS) method for statistical analysis.

### 3.3. Procedure

The measuring approach was employed in the first phase to assess the constructs’ validity, convergence, and reliability. This involved assessing the reliability of the assessment questions, proving convergent validity with “factor loadings and average variance extracted (AVE)”, and showing “discriminant validity using cross-loadings and the Fornell-Larcker criteria”. In order to evaluate the hypotheses, structural equation modelling (SEM), was then applied in the second step. To evaluate the latent constructs’ discriminant validity, convergence, and reliability, the measurement model of the constructs was determined at first.

#### 3.3.1. Statistical analysis

**Fig 1:**
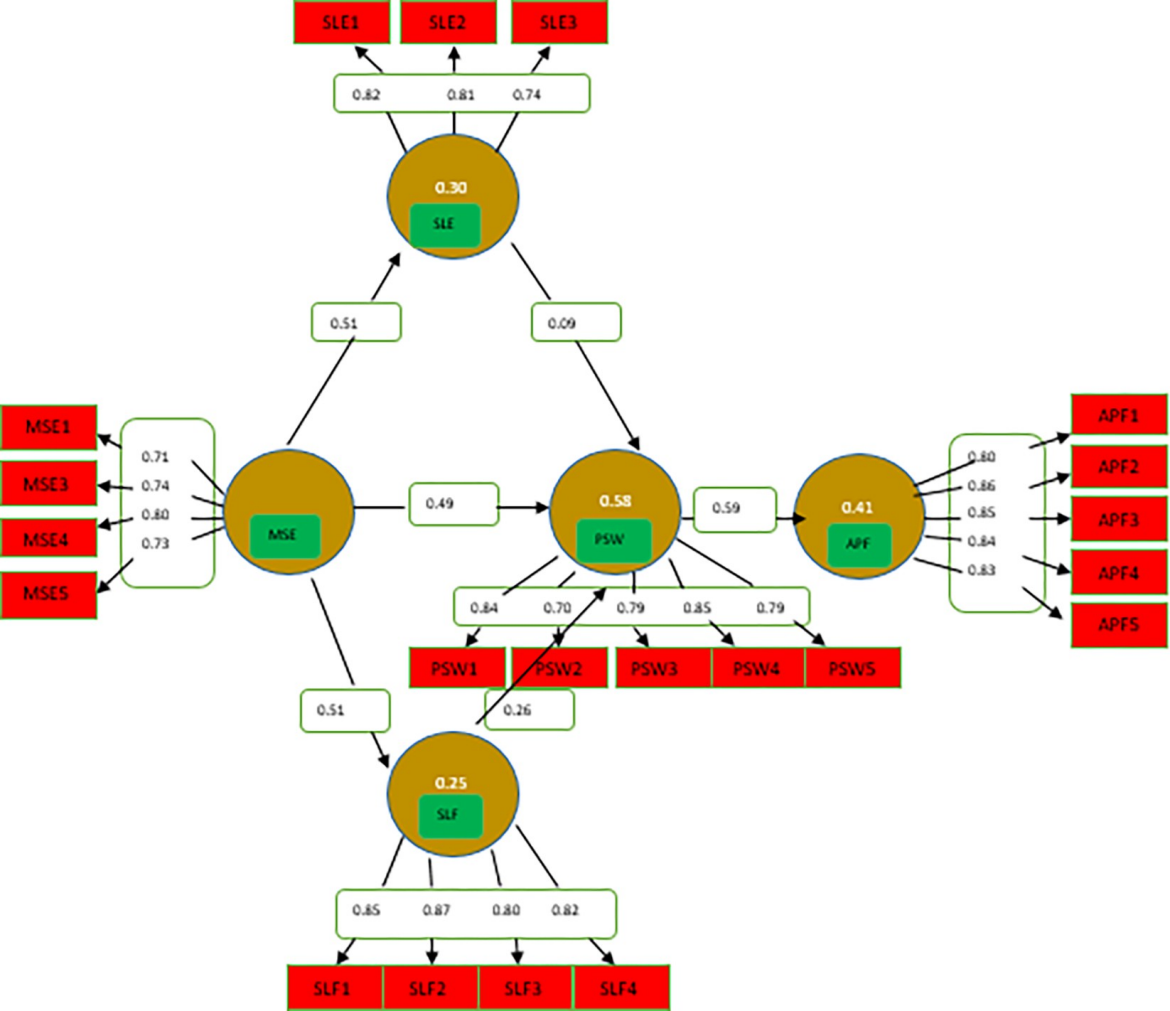
Measurement model. Note: “MSE = music education; PSW = psychological well-being; SLE = self-esteem; Self-efficacy = SLF; APF = Academic performance”.

In a reflective model, when the indicators are impacted by the latent variable, the measuring model shows arrows from the latent variables heading towards the indicators, as seen in Fig 1.

A construct is deemed acceptable if its outer loading value exceeds 0.70, meaning that it captures 70% or more of its components [33, 73]. Some other research suggests the value of more than 0.50 is also appropriate [74]. All outside loadings in this investigation were more than 0.50, as shown in Table 2, indicating reliability.

**Table 2:** Reliability and validity.

Construct	Items/Elements	Outer Loading	Alpha	rho-A	CR	AVE
**Psychological/Mental Well-Being**			0.84	0.85	0.89	0.63
	PSW1	0.82				
	PSW2	0.70				
	PSW3	0.76				
	PSW4	0.81				
	PSW5	0.80				
**Self Esteem**			0.82	0.81	0.86	0.65
	SLE1	0.76				
	SLE2	0.80				
	SLE3	0.72				
**Self Efficacy**			0.90	0.91	0.92	0.70
	SLF1	0.83				
	SLF2	0.85				
	SLF3	0.80				
	SLF4	0.81				
**Academic Performance**						
	APF1	0.80				
	APF2	0.85				
	APF3	0.88				
	APF4	0.81				
	APF5	0.85				
**Music Education**			0.75	0.77	0.79	0.72
	MSE1	0.77				
	MSE3	0.76				
	MSE4	0.80				
	MSE5	0.70				

“CR = Cronbach’s alpha; CA = composite reliability; AVE = average variance extracted; MSE = music education; PSW = psychological well-being; SLE = self-esteem; Self-efficacy = SLF; APF = Academic performance”

To improve the outcomes, however, elements MSE2, SLE4, SLF5, and PSW6 were eliminated from the model. The internal consistency of the constructs was evaluated using rho_A test, compound reliability, and Cronbach’s alpha. To find if constructs are internally consistent, Cronbach’s alpha must be greater than 0.7 [73]. Table 2 demonstrates that all Cronbach’s alpha values are greater than 0.70, showing good internal consistency. Composite reliability (CR) is an additional technique for evaluating a scale’s internal consistency and dependability. The studies [68, 73, 75] state that internal consistency is shown by CR values more than 0.70. It is shown that all underlying hypotheses have composite reliability values greater than 0.70, indicating internal consistency. Furthermore, it is [68, 73, 76] recommended that rho_A values larger than 0.70 be used to determine reliability. All of the rho_A values are more than 0.70, which further supports internal consistency. According to Hair et al. [77], convergent validity describes how closely all of the components within a concept are connected to one another. An AVE threshold value of 0.5 or above [73], indicates convergent validity.

### 3.4. Discriminant validity

The term "discriminant validity" describes how one construct differs experimentally from the others. It guarantees that every latent variable in the model is unique. This study used Fornell and Larcker criteria to evaluate the discriminant validity. “According to this criteria, each variable’s square root of its Average Variance Extracted (AVE) should be larger than its correlations with other variables in order to demonstrate discriminant validity” [68]. Table 3 shows that for every build, the square root of the AVE is greater than the diagonal value connections with other constructs, confirming the discriminant validity.

**Table 3:** Fornell and larker.

	MSE	PSW	SLE	SLF	APF
**MSE**	**0.77**				
**PSW**	0.69	**0.80**			
**SLE**	0.46	0.52	**0.81**		
**SLF**	0.49	0.58	0.50	**0.85**	
**APF**	0.48	0.61	0.49	0.48	**0.83**

### 3.5. Collinearity statistics (VIF)

Values of the variance inflation factor (VIF) are used in structural models to evaluate possible common method bias and collinearity problems. According to the study [73], the values of VIF would be less than three in order to find significance. It functions as an indicator to ascertain whether indicator variables are associated with each other or not. According to this research, every VIF number, as shown in Table 4, is less than three. Consequently, we draw the conclusion that the variables in the suggested structural model do not have any collinearity problems.

**Table 4:** Collinearity statistics (VIF).

Construct	VIF
MSE1	1.42
MSE3	1.52
MSE4	1.72
MSE5	1.58
PSW1	2.01
PSW2	1.52
PSW3	1.76
PSW4	2.48
PSW5	1.68
SLE1	1.35
SLE2	1.52
SLE3	1.36
SLF1	2.00
SLF2	2.17
SLF3	1.59
SLF4	1.69
APF1	1.85
APF2	2.04
APF3	2.12
APF4	2.25
APF5	2.16

Before running the structural evaluation model, the model fitting parameters were assessed. SRMR *(Standardized Root Mean Square Residual)* and NFI *(Normed Fit Index)* are two often used model fit parameters. SRMR measures the discrepancy between the correlation matrix produced by the model and the actual correlation, ideally with values less than 0.08 [78]. The SRMR value in this study is more than 0.08, which is insufficient to fulfill the requirements. Ideally, NFI, the second model fit parameter, should be higher than 0.90. The normed fit index value in this study is 0.901, satisfying the acceptable requirement.

The SEM determines the relation between the independent and dependent variables. The degree to which the independent variables can account for the variance in the dependent variable is indicated by the coefficient of determination (R^2^). “R^2^ values of 0.75, 0.50, and 0.25 are regarded as significant, moderate, and weak, respectively” [73]. The R^2^ values of all variables are reported in the Table 5.

**Table 5:** R^2^.

	R^2^	SD	T-Value	P-Value
**PSW**	0.55	0.05	10.53	0.00
**SLE**	0.24	0.04	3.86	0.00
**SLF**	0.28	0.06	4.76	0.00
**APF**	0.42	0.05	7.63	0.00

### 3.6. Hypothesis testing

The first hypothesis reveals the relation among psychological well-being and music learning. The findings show that psychological well-being is significantly and favorably impacted by music education. It is accepted that this hypothesis since the p-value is less than 0.05. The second hypothesis shows the relation among self-esteem and music education. The findings demonstrate that self-esteem is significantly and favorably impacted by music education. A link between self-efficacy and music education was suggested by hypothesis 3. The findings show that self-efficacy is significantly and favorably impacted by music education. A connection between psychological wellness and self-esteem was suggested by hypothesis 4. The findings indicate that psychological wellness is significantly and favorably impacted by self-esteem. A connection between psychological wellness and self-efficacy was suggested by hypothesis 5. The empirical findings of hypotheses are shown in the following Table 6.

**Table 6:** Hypothesis testing.

Hypotheses	β	SD	T-Value	P-Value	Decision
***H1***	0.55	0.052	8.12	0.01	*Supported*
***H2***	0.52	0.053	7.83	0.00	*Supported*
***H3***	0.50	0.04	9.47	0.02	*Supported*
***H4***	0.15	0.07	3.35	0.01	*Supported*
***H5***	0.32	0.09	4.32	0.03	*Supported*
***H6***	0.15	0.03	2.38	0.02	*Supported*
***H7***	0.18	0.04	3.83	0.00	*Supported*
***H8***	0.58	0.05	8.28	0.00	*Supported*

The findings show that psychological wellness is significantly and favorably impacted by self-efficacy. According to Hypothesis 8, psychological wellbeing significantly improves academic performance of students. According to the findings, psychological well-being significantly and favorably affects students’ academic performance. According to hypothesis six, the association between psychological well-being and music education is mediated by self-esteem. The outcomes show that self-esteem positively and significantly mediates this relationship. The study hypothesized in *H7* that self-efficacy acts as a mediator in the relationship between psychological well-being and music education. The findings confirm that self-efficacy positively mediates this association significantly.

## 4. Discussion

This study was conducted with a special focus on goals to evaluate how music education affected students’ academic performance and psychological health. The first three hypothesis indicate are supported that music, self-esteem and self-efficacy improve the students’ psychological well-being. These findings align with a number of earlier research projects, such as those conducted by the studies on the topic [39, 70, 71, 79]. The results of this study support the well-documented relationship between music education and enhanced psychological well-being among students. Similar to findings of the study [52], this study indicates that students engaged in music learning report higher levels of psychological well-being, which includes factors such as reduced anxiety, improved mood, and greater life satisfaction. Krause and colleagues utilized Self-Determination Theory to explain how musical participation fosters intrinsic motivation and social connectedness, which in turn enhances well-being. Moreover, another study [62] provided a comprehensive review of how music practice contributes to psychological well-being by influencing positive emotions, engagement, and a sense of accomplishment. It is highlighted that the importance of emotional regulation through music, this suggests that the benefits of music education are not only emotional but also cognitive, as students develop confidence in their abilities, which positively influences their overall psychological health. On the other hand, the study [49] found a strong association between music education and happiness, particularly among university music students. This indicates that while music education directly enhances happiness, it also does so indirectly by fostering a sense of self-worth and personal competence. This adds depth to the understanding of how music education impacts psychological well-being by showing that its benefits are multifaceted and operate through both direct and indirect pathways. In contrast to some studies that primarily emphasize the social aspects of music education, it is also found that group music activities improve social skills and collective well-being [26, 47, 51, 72]. While social factors are undoubtedly important, the findings suggest that personal, internal growth facilitated by music education is equally crucial in improving students’ psychological well-being. The findings also confirm the positive relation among music education and improvements in self-esteem and self-efficacy among students, consistent with findings of earlier research. For instance, the study [27] observed that “children who received piano instruction over three years exhibited significant increases in self-esteem compared to those who did not receive such instruction”. Similarly, this study found that students engaged in music learning, whether through instrumental or vocal training, demonstrated higher levels of self-esteem, suggesting that music education serves as a powerful tool for enhancing students’ self-perception and confidence. Another study [54] investigated the self-efficacy among music students and found that mastery experiences, such as successfully learning a piece of music, played a critical role in boosting students’ self-efficacy. This study confirms these findings by showing that music education contributes significantly to students’ self-efficacy, particularly through the development of personal competence and the accomplishment of musical tasks. Moreover, Rashid and Zaman [73] emphasized the importance of teachers’ behavior in influencing students’ academic performance and self-efficacy, highlighting that supportive and encouraging music educators can foster a positive learning environment that enhances students’ belief in their abilities. This study builds on this by showing that beyond teacher influence, the intrinsic aspects of music education itself—such as the personal achievements and mastery it promotes—are crucial for developing self-efficacy. This suggests that while external factors like teacher support are important, the internal experiences provided by music education are equally critical for boosting self-efficacy. While these studies largely focused on either self-esteem or self-efficacy individually, this research is unique in exploring both constructs simultaneously and identifying their interrelatedness. Self-Determination Theory posits that self-esteem and self-efficacy are closely linked, with both being essential for optimal psychological development [38, 73]. Our findings support this theory by demonstrating that music education positively impacts both self-esteem and self-efficacy, which together contribute to students’ overall psychological well-being. This dual focus provides a more comprehensive understanding of how music education influences personal development. Furthermore, Wu and Lu [46] examined the “role of musical training in the development of empathy and prosocial behaviors, linking these traits to increased self-esteem and self-efficacy”. While their study highlighted the social aspects of music education, our research emphasizes the internal processes—particularly “how self-esteem and self-efficacy mediate the relationship between music education and psychological well-being”. This distinction suggests that while music education enhances social skills, it also fundamentally strengthens personal beliefs and self-worth, which are critical for students’ long-term success and mental health.

It can be argued that “music provides a platform for students to express their emotions. Through playing instruments or singing, students can convey feelings that might be difficult to express verbally, leading to a sense of emotional release and relief. Moreover, engaging with music, whether by listening, playing, or composing, has been shown to reduce stress levels. It can lower cortisol levels (a stress hormone) and increase the production of endorphins, which are chemicals in the brain that promote a sense of well-being” [44]. In addition, music has the power to uplift moods and evoke positive emotions. Learning to play an instrument or singing in a choir can create a sense of accomplishment and joy, which can improve overall mood and well-being. It is also argued that “music education has been associated with improved cognitive function, including enhanced memory, attention, and executive functions” [62]. These benefits can contribute to overall psychological well-being. Music education often involves group activities such as playing in an ensemble or performing in a choir. These experiences foster social connections and a sense of belonging, which are important for psychological well-being. These insights have practical implications for educators and policymakers. Integrating music education into the curriculum could be a strategic way to enhance students’ self-esteem and self-efficacy, thereby promoting their overall psychological development. Future research could further explore how different types of music education (e.g., individual vs. group lessons) differentially impact self-esteem and self-efficacy, as well as the long-term effects of these improvements on students’ academic and personal success.

Early research [46–48, 74] have posited that music education enhances students’ psychological well-being and academic achievement by elevating their self-efficacy and self-esteem. This was discussed before in the section on the literature review. Our results support previous studies and demonstrate the importance of music in fostering students’ sense of competence and self-esteem. The study’s findings corroborated the fourth hypothesis, which examined the connection between students’ psychological health and self-esteem. This correlation may be explained by the fact that high self-esteem is necessary for fostering a strong sense of confidence, which in turn benefits students’ psychological health [57, 75]. These results align with a number of other studies [48, 49], which presented similar results from other perspectives. Similar results were obtained when inspecting the association between learners’ psychological wellness and self-efficacy. This is because students’ psychological wellbeing, is influenced by their self-efficacy, which is linked to their self-esteem. These judgments are reliable with earlier studies [55, 56, 68]. The results of this study are in line with those of studies conducted by the studies [57, 59, 76], which looked at the mediating effects of students’ self-efficacy and self-esteem in similar ways. The results of this study indicate that self-efficacy and self-esteem serve as significant mediators in the relationship between music education and psychological well-being. This finding aligns with and expands upon previous research in several key ways. Previous research, such as [46] suggested that arts-based programs, including music education, enhance students’ psychological well-being by boosting their self-esteem. In their study, self-esteem was found to be a crucial factor that mediated the positive effects of music education on overall mental health. It is shown that students who engage in music learning experience increased self-esteem. This supports the theory that self-esteem acts as a bridge between the skills and accomplishments gained through music education and the broader sense of well-being. Moreover, Rickard et al. [48] examined the impact of sustained music education on adolescent self-esteem and found that consistent engagement with music led to long-term improvements in self-esteem. Our study not only confirms these findings but also highlights self-esteem’s role as a mediator, emphasizing that the psychological benefits of music education are partly realized through enhanced self-worth. Zelenak [44], identified that music students often developed a heightened sense of self-efficacy through mastery experiences. This increased self-efficacy contributed to overall psychological well-being by fostering a sense of competence and control. Our study builds on these findings by demonstrating that self-efficacy not only improves as a result of music education but also mediates its impact on well-being. This suggests that the confidence students gain from musical achievements is a key factor in translating music education into psychological benefits. Additionally, the study [51] explored the relation among self-efficacy and music performance, noting that students with higher self-efficacy succeed and feel positive about their musical experiences. Our study extends this by showing that the benefits of increased self-efficacy are not limited to music performance alone but also extend to general psychological well-being, underscoring the importance of self-efficacy as a mediator. While previous studies have often focused on either self-esteem or self-efficacy as mediators, our study is unique in examining both constructs simultaneously. Ryan and Deci [36], through Self-Determination Theory, posited that self-esteem and self-efficacy are interrelated and crucial for psychological health. Our findings support this theory by showing that “both self-esteem and self-efficacy jointly mediate the relationship between music education and psychological well-being. This dual mediation suggests that the interplay between self-efficacy and self-esteem is essential for fully understanding how music education enhances mental health” [37]. These findings have important implications for educators and policymakers. Recognizing the mediating roles of self-efficacy and self-esteem can inform the design of music education programs that explicitly aim to boost these constructs, thereby enhancing students’ overall well-being. Future research could further explore the relative strength of each mediator and investigate how different forms of music education might differentially impact self-efficacy, self-esteem, and well-being.

The last hypothesis looked at the linkage among academic achievement and students’ mental health. It is often known that students do better academically and get higher marks or academic accomplishment when they are mentally engaged, effective, and have good mental health. Consistent outcomes from this hypothesis supported the conclusions of earlier studies [65–66, 72, 77]. The study’s findings provide compelling evidence for the value of integrating music education into schools in order to improve children’ psychological health and, eventually, their academic achievement. The findings of this study indicate a significant positive relationship between psychological well-being and academic performance among students. This relationship has been widely explored in previous research, and our findings align with, extend, and offer new insights into this well-established connection. Several studies have consistently demonstrated that students with higher levels of psychological well-being tend to perform better academically. For instance, the study [53, 78] found that students with greater life satisfaction, a key component of psychological well-being, showed superior academic outcomes compared to their less satisfied peers. This study confirms these findings, reinforcing the idea that psychological well-being acts as a foundational element that supports academic success. This relationship likely stems from the fact that well-being enhances cognitive functioning, motivation, and engagement in school-related activities. Similarly, another study [44, 80] highlighted that psychological well-being, including positive emotions and life satisfaction, is linked to higher grades and better overall academic achievement. Our research aligns with this by showing that students with higher psychological well-being are more likely to excel academically, suggesting that interventions aimed at improving well-being could also lead to better academic outcomes. While previous studies have generally linked overall psychological well-being to academic performance, our study delves deeper into specific dimensions of well-being, such as emotional stability and social connectedness, and their individual impacts on academic outcomes. It argued that positive affect and emotional stability are crucial for optimal functioning in academic settings [29]. The findings support this by demonstrating that students who experience emotional stability, a key aspect of psychological well-being, tend to have better focus, resilience, and academic performance. Moreover, the study [51, 81] emphasized the role of social connectedness—a component of psychological well-being—in enhancing academic achievement. This suggests that fostering a sense of belonging and social support within educational settings could be a strategic way to improve both well-being and academic outcomes. This study contributes new insights by examining not only the direct relationship between psychological well-being and academic performance but also the potential mediating factors, such as self-efficacy and self-esteem. This approach underscores the importance of a supportive psychological environment, where both well-being and self-efficacy are nurtured to optimize academic outcomes. Furthermore, this study suggests that the impact of psychological well-being on academic performance may be cumulative over time, with sustained well-being leading to progressively better academic results, highlighting the importance of long-term strategies for enhancing student well-being. The findings of this study and previous research suggest that educational institutions should prioritize psychological well-being as part of their academic strategy. Programs that promote emotional stability, social connectedness, and overall mental health could lead to substantial improvements in academic performance. Future research could explore the effectiveness of specific interventions aimed at enhancing well-being and track their impact on academic outcomes over time.

### 4.1. Theoretical implications

This research work adds significantly to the body of literature in a number of ways. In the first place, it fills a vacuum in the literature by examining the origins and consequences of students’ psychological and mental health. It is imperative to investigate this area and offer workable ways to reduce stress, anxiety, and burnout among graduate and undergraduate students. The study’s findings show how important music education is for raising psychological well-being and, subsequently, academic performance of students. This study conceptualizes a process by which music teaching influences student outcomes by integrating concepts from social cognitive theory, self-determination theory, and self-esteem theory. To be more precise, the outcomes indicate that music learning helps the students to become more confident and self-assured, both of which are beneficial to their psychological health. This means that adding music education to academic curricula can be a useful strategy for supporting students’ mental health and academic achievement.

### 4.2. Practical implications

The study’s conclusions have significant ramifications for educators and politicians. They emphasize how important it is to include music education in undergraduate and graduate programs since it has a lasting positive effect on students’ psychological health. Thus, it is imperative that the integration of music education within academic curriculum be given top priority. Furthermore, the findings highlight how important self-efficacy and self-esteem are in improving students’ psychological well-being. As a result, educational institutions ought to give top priority to developing students’ feeling of self-esteem and self-efficacy through their curricula, training courses, and other activities. In addition, it is critical that educational establishments give instructors the training they need to deal with students’ anxiety, stress, and depression. These mental health issues frequently pose serious obstacles to students’ performance in the classroom and in the workplace. Academic institutions may foster a more positive learning environment that supports students’ overall welfare and success by providing instructors with the skills and tools they need to help students’ mental health. The findings of this study also have significant implications for educational policies and practices, highlighting the importance of integrating music education into school curricula to promote students’ psychological development, self-esteem, and self-efficacy. The results can inform resource allocation, teacher training, and assessment methods, emphasizing the value of music learning beyond academic achievement. The study’s findings can also support inclusive education policies, student well-being initiatives, and curriculum design, ultimately contributing to a more comprehensive and supportive learning environment. By acknowledging the psychological benefits of music learning, educators and policymakers can work together to create a more holistic educational experience that fosters students’ overall well-being and success.

### 4.3. Limitations and future research

Notwithstanding the noteworthy contributions of this investigation, it is imperative to recognize a few restrictions in order to augment the resilience of subsequent research undertakings. First off, sample bias may have been incorporated into this study due to the convenience sampling method used. More exacting sampling strategies would be used in upcoming research to guarantee the generalizability of results. Second, longitudinal or time-series approaches may be used in future research to capture changes over time and produce stronger evidence. Furthermore, there could be other factors that affect the association between music education and psychological wellness, even though this study concentrated on the mediating roles of self-esteem and self-efficacy. Future studies have to look at other factors that could improve students’ psychological welfare.

## 5. Conclusions

Music education is recognized as an essential instrument for reducing students’ stress and anxiety, which affects their academic performance and personal growth. The main objective of the study is to assess the impact of music learning on academic performance and psychological well-being of students. The study also intended to investigate how self-efficacy and self-esteem act as mediators in relation of music education and psychological well-being. To explain, this study used a theoretical framework derived from the theories of social cognition, self-esteem, and self-efficacy. Utilizing a quantitative method, convenience sampling was employed to gather the data from 326 undergraduate and graduate students via self-administered questionnaires and applied PLS-SEM for empirical analysis. The findings of the study reveal that music education significantly affects the academic performance and psychological well-being of students. Furthermore, it is shown that self-efficacy and self-esteem play their role as mediators between psychological well-being and music education. These findings pave the way for future studies by offering insightful information in literature of music education and psychological well-being. It is recommended that policymakers and practitioners should advocate the inclusion of music education in order to improve the educational and psychological well-being of students.

## Supporting information

S1 Data (XLSX)
